# Improving access to healthcare for paediatric sickle cell disease patients: a qualitative study on healthcare professionals’ views

**DOI:** 10.1186/s12913-021-06245-2

**Published:** 2021-03-12

**Authors:** Maite E. Houwing, Marit Buddenbaum, Thijs C. J. Verheul, Anne P. J. de Pagter, Jacobus N. J. Philipsen, Jan A. Hazelzet, Marjon H. Cnossen

**Affiliations:** 1 Department of Pediatric Haematology, Erasmus University Medical Centre – Sophia Children’s Hospital, Wytemaweg 80, 3015 CN Rotterdam, The Netherlands; 2 Department of Cell Biology, Erasmus University Medical Centre, Rotterdam, The Netherlands; 3 Department of Public Health, Erasmus University Medical Centre, Rotterdam, The Netherlands

**Keywords:** Sickle cell disease, Access to health care, Accessibility of health services, Qualitative research, Health care professionals

## Abstract

**Background:**

In well-resourced countries, comprehensive care programs have increased life expectancy of patients with sickle cell disease, with almost all infants surviving into adulthood. However, families affected by sickle cell disease are more likely to be economically disenfranchised because of their racial or ethnic minority status. As every individual child has the right to the highest attainable standard of health under the United Nations Convention on the Rights of the Child, it is essential to identify both barriers and facilitators with regard to the delivery of adequate healthcare. Optimal healthcare accessibility will improve healthcare outcomes for children with sickle cell disease and their families. Healthcare professionals in the field of sickle cell care have first-hand experience of the barriers that patients encounter when it comes to effective care. We therefore hypothesised that these medical professionals have a clear picture of what is necessary to overcome these barriers and which facilitators will be most feasible. Therefore, this study aims to map best practises and lessons learnt in order to attain more optimal healthcare accessibility for paediatric patients with sickle cell disease and their families.

**Methods:**

Healthcare professionals working with young patients with sickle cell disease were recruited for semi-structured interviews. An interview guide was used to ensure the four healthcare accessibility dimensions were covered. The interviews were transcribed and coded. Based on field notes, initial codes were generated, to collate data (both barriers and solutions) to main themes (such as “transportation”, or “telecommunication”). Through ongoing thematic analysis, definitive themes were formulated and best practices were reported as recommendations. Quotations were selected to highlight or illustrate the themes and link the reported results to the empirical data.

**Results:**

In 2019, 22 healthcare professionals from five different university hospitals in the Netherlands were interviewed. Participants included (paediatric) haematologists, nurses and allied health professionals. Six themes emerged, all associated with best practices on topics related to the improvement of healthcare accessibility for children with sickle cell disease and their families. Firstly, the full reimbursement of invisible costs made by caregivers. Secondly, clustering of healthcare appointments on the same day to help patients seeing all required specialists without having to visit the hospital frequently. Thirdly, organisation of care according to shared care principles to deliver specialised services as close as possible to the patient’s home without compromising quality. Fourthly, optimising verbal and written communication methods with special consideration for families with language barriers, low literacy skills, or both. Fifthly, improving the use of eHealth services tailored to users’ health literacy skills, including accessible mobile telephone contact between healthcare professionals and caregivers of children with sickle cell disease. Finally, increasing knowledge and interest in sickle cell disease among key stakeholders and the public to ensure that preventive and acute healthcare measures are understood and safeguarded in all settings.

**Conclusion:**

This qualitative study describes the views of healthcare professionals on overcoming barriers of healthcare accessibility that arise from the intersecting vulnerabilities faced by patients with sickle cell disease and their families. The recommendations gathered in this report provide high-income countries with a practical resource to meet their obligations towards individual children under the United Nations Convention on the Rights of the Child.

## Background

### Sickle cell disease: a global health challenge

Sickle cell disease (SCD) is an autosomal, recessive haemoglobinopathy characterised by ongoing haemolytic anaemia, episodes of acute pain caused by vaso-occlusion (vaso-occlusive crises), and progressive organ failure. It is the most common monogenetic disease worldwide with an estimated 300,000 births annually and is recognised as an important public health problem by the World Health Organisation (WHO) [1].

Seventy-five per cent of the global burden of SCD occurs in sub-Saharan Africa, where the majority of children with the disease do not reach their fifth birthday [2]. In contrast, the life expectancy in well-resourced countries has significantly improved with almost all infants now expecting to survive into adulthood due to comprehensive care programs. However, the life expectancy of patients with SCD is still 20–30 years shorter than the average life span of the general population [3].

The Netherlands currently counts approximately 1500 SCD patients, half of which are children [4]. Most of those affected are from Asian or African ancestry, with a minority being of Middle Eastern descent [5]. In the Netherlands, care for paediatric patients with SCD is organised in centralised, comprehensive care centres, to ensure good quality of care [6].

### Vulnerabilities in sickle cell disease

In western countries, SCD predominantly affects racial and ethnic minorities. It is well-known that children from non-western ethnic minorities are more likely to live in poverty and reside in families with a lower family income [7]. Low socioeconomic status is associated with higher rates of illness, shorter life expectancy, high stress levels, low birth weight and many other negative health outcomes [8]. In addition to socioeconomic disadvantage, children with SCD and their families encounter many psychosocial issues including increased anxiety, depression, social withdrawal, aggression, poor relationships, poor school performance and impaired health-related quality of life [9–12]. These psychosocial issues mainly result from the impact of pain and other disease symptoms on everyday life, however they are also a result of society’s unawareness of SCD and the lack of understanding and empathy towards those affected. Although life expectancy has improved, many outcome goals remain unmet. This is not only due to the biological burden of acute complications or chronic morbidity such as multiorgan failure, but also due to the complex interaction between patients with SCD and the socioecological system [13–15]. SCD has historically been described as a “black disease” [16]. This harmful association of the disease with race has resulted in social and ethical consequences that are tied to discrimination [15]. For example, the pain complaints of racial minorities are less likely to receive adequate attention due to the often complex communication between the patient and physicians or nurses [17–20]. In addition to stigmatisation in healthcare, significant gaps exist in both the equity of research funding and philanthropy for SCD [21, 22].

### Evaluating access to healthcare

The accessibility of healthcare concerns the level at which people are able to utilise all healthcare resources they need to sustain or improve their health [23]. This accessibility is described by four overlapping dimensions: physical accessibility of healthcare, affordability of healthcare, accessibility of health-related information, and the principle of non-discrimination [24, 25]. The comparison of ‘amenable mortality’ rates between countries allows the approximation of national levels of healthcare access and quality [26]. The Netherlands ranked 3rd in the Healthcare Access and Quality (HAQ) Index of the 2016 Global Burden of Disease Study [26]. From a global perspective, accessibility of healthcare might therefore not be seen as a matter of concern for Dutch clinical practice. The evaluation of the HAQ Index however, provides limited insight in accessibility disparities between different groups of society, or between patients with different diseases. Healthcare professionals have first-hand experience of barriers faced by patients when it comes to effective care. From professionals’ anecdotal and seemingly unique stories, a picture emerges of the general challenges faced by our healthcare system when it aims to provide access to the highest attainable standard of care to every individual.

### Access to the highest attainable standard of healthcare

Like most countries in the world, the Netherlands has signed and ratified multiple human rights treaties and conventions. The commitments made in these documents are important in the context of healthcare for children. As early as 1966, the International Covenant on Economic, Social and Cultural Rights (ICESCR) established access to healthcare as a fundamental human right. Furthermore, the Committee on Economic, Social and Cultural Rights (CESCR) obligates states parties to ensure equality in the access to healthcare and health-related services. They emphasise that children should be regarded as a vulnerable group that require explicit protection [27]. In 1989, the United Nations Convention on the Rights of the Child (UNCRC) reinforced “the right of the child to the enjoyment of the highest attainable standard of health” to provide additional safeguards for the protection of children [28]. In 2019, the UNCRC celebrated its 30th anniversary and to commemorate this milestone, we aimed to assess its implementation in a high-income country, focussing on accessibility of care for paediatric patients with SCD in the Netherlands as a case study.

### Study aim

In this nationwide assessment, we aim to map barriers and facilitators in any of the four dimensions of healthcare accessibility faced by Dutch children with SCD and their families. By interviewing healthcare professionals, we try to identify common challenges and lessons learnt in clinical practice on a grassroots level.

## Methods

### Design and study setting

In this study, a qualitative descriptive design was used. The qualitative approach, with its focus on subjective experience, is best to enhance understanding of the range of problems with healthcare accessibility that patients experience and that healthcare professionals observe. Interviews were conducted with SCD healthcare professionals working in various care settings. Participants were affiliated with the ‘SCORE’ (Sickle Cell Outcome Research) consortium of the Netherlands which includes all SCD comprehensive care centres and research institutes involved in clinical SCD research in the Netherlands. Study findings are reported in accordance with the Standards for Reporting Qualitative Research (SRQR) [29]. This project was approved by the Medical Research Ethics Committee of Erasmus University Medical Center and adhered to the Declaration of Helsinki [30]. The participants provided written informed consent.

### Participants

To recruit a purposeful sample, we sought healthcare professionals, including (paediatric) haematologists, nurse practitioners, nurses, psychosocial staff and social workers, who provided care to paediatric and adolescent SCD patients. We identified eligible participants through a central list of SCORE professionals (key informant sample) and recruited a broad range of participants using a combination of maximum variation and snowball sampling [31, 32]. A cyclical approach to sampling, conducting interviews and analysis and interpretation, allowed theoretical saturation to be attained when no new themes – related to healthcare accessibility – emerged from subsequent interviews [32].

### Data collection

Three trained investigators (M.E.H., M.B. and T.C.J.V.) conducted face-to-face semi-structured, in-depth interviews between February 12th and May 23rd, 2019. One week before the interview, each healthcare professional received an e-mail explaining the purpose of the study and our specific interest in access to healthcare for children and adolescents with SCD. Interview questions were formulated to probe the healthcare professionals to elaborate and explain the challenges faced by their patients and to provide recommendations on how to solve these issues. An interview guide was used to ensure the four healthcare accessibility dimensions were covered and started with the question of how the participant would define access to health. The interview guide contained only open questions aiming to freely explore the participants’ experience. Two examples of these questions (in this case focussed on the accessibility of information) were:

- What do you aim for when informing patients?
- What happens after you have informed the patients?

The participants’ initial response was often followed by a probing question, such as:

- Could you give an example?
- How is this different for patients with SCD compared to other patient groups?

The Interviews occurred privately at the workplace of the participants. Interviews were conducted in Dutch and were audio-recorded. Field notes with initial thought were made by the interviewers after each interview.

### Data analysis

The interviews were transcribed verbatim and coded, followed by a thematic analysis [33]. Based on field notes, initial codes were generated to collate data (both problems and solutions) to main themes (such as “transportation”, or “telecommunication”). Through ongoing thematic analysis, definitive themes were formulated. The transcripts were analysed by three researchers (M.E.H., M.B. and T.C.J.V.). The results (transcripts, codes and themes) were subsequently discussed with experts in the field of healthcare accessibility or the field of clinical paediatric sickle cell care to confirm the accuracy of the analyses. The robustness of the research was increased by selecting quotations to highlight or illustrate the themes and link the reported results to the empirical data. To increase readability for the general public, the definitive themes have been reported as recommendations.

## Results

### Sample description

**Fig. 1:**
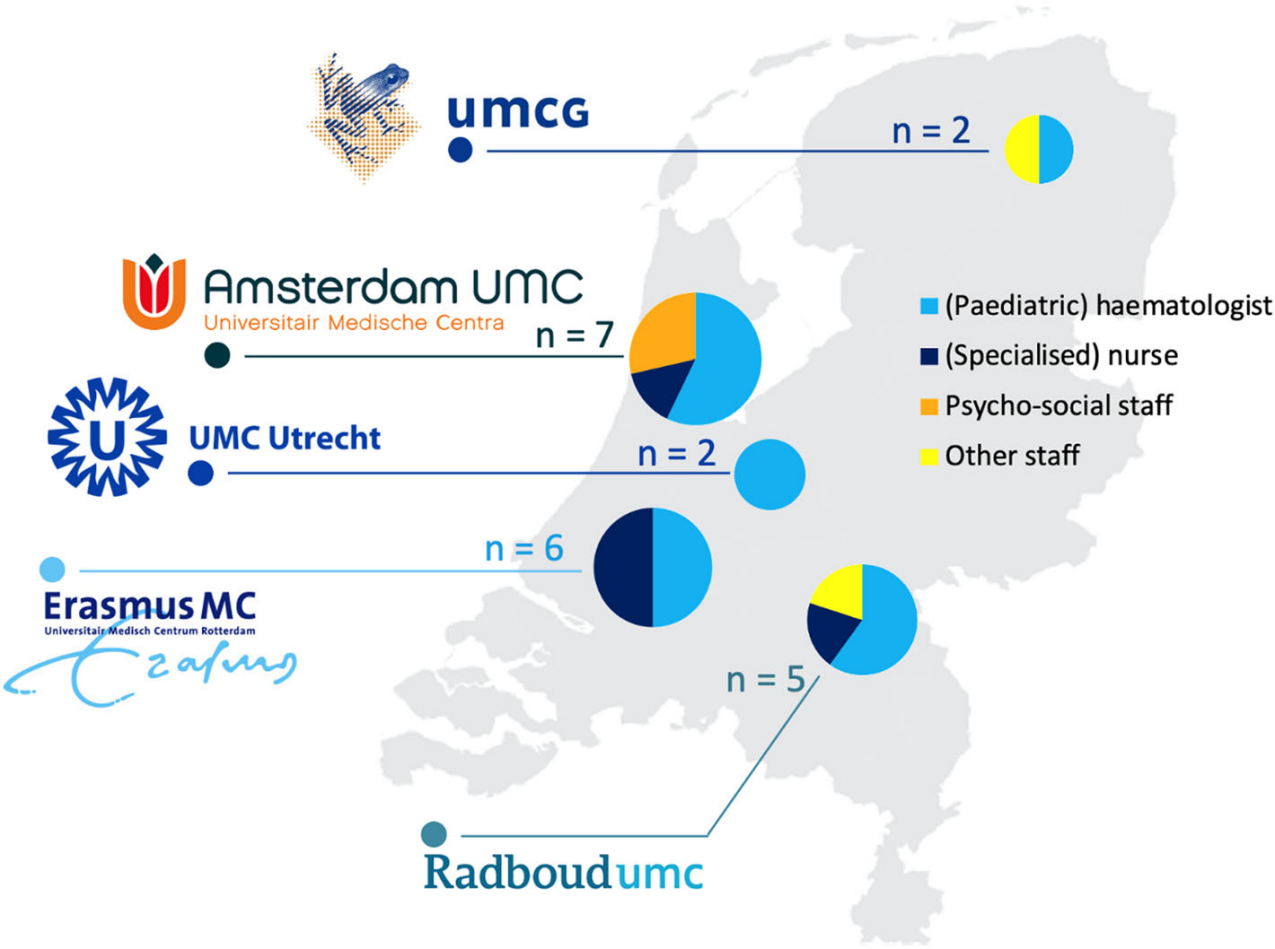
Sickle cell disease comprehensive care centres of the twenty-two interviewed participants

Twenty-two healthcare professionals from five different academic clinic sites for comprehensive sickle cell care in the Netherlands participated in the study (Fig. 1). None of the potential participants declined to participate in the study. The participants’ mean age was 37.0 (SD 14.5) years. Of the 22 participants, 19 were women and 21 where white. The average number of years of experience in their profession was 8.5 (SD 6.5). Interviews lasted on average 38 min (range: 15 to 58 min). Table 1 summarises study participant characteristics.

**Table 1:** Participant characteristics

Item	Number (n)	Percentage (%)
**Sex**		
**Male**	3	87
**Female**	19	13
**Mean Age (years)**	37.0	NA
**Ethnicity**		
**White Dutch**	21	96
**Black (Dutch Caribbean)**	1	4
**Provider type**		
**(Paediatric) haematologist**	13	59
**(Specialised) nurse**	5	23
**Psycho-social staff**	3	14
**Other staff**	1	4
**Years of experience in sickle cell care**		
**< 5 years**	7	32
**5–10 years**	6	27
**> 10 years**	9	41
**Clinical centre**		
**Erasmus University Medical Centre Rotterdam**	6	27
**Amsterdam University Medical Centres**	7	32
**Radboud University Medical Centre**	5	23
**University Medical Centre Utrecht**	2	9
**University Medical Centre Groningen**	2	9

*Abbreviations*: *NA* not applicable

Thematic analyses of the interview transcripts, revealed six themes, or recommendations, on how to improve healthcare accessibility for children with SCD and their families.

### Theme 1. Cutting invisible costs: addressing the financial burden of a child with sickle cell disease

In general, Western countries provide free public healthcare insurance for children to ensure healthcare access for everyone under 18 years of age. However, depending on countries and healthcare systems, some medical services are subject to a statutory personal contribution. In addition, direct nonmedical costs (i.e. travel expenditures and telephone calls to the hospital) and indirect costs (i.e. missed workdays for caregivers and childcare for siblings) are generally not reimbursed. Many participants reported that families had difficulties with costs.

*“Last month, we had a seven-year-old visiting our outpatient clinic on his own. We asked where his mom or dad was. ‘In the car’ he replied. His mother didn’t have enough money to pay for the relatively high hospital parking fee.”*

Participants felt that the government or insurance companies should ensure that caregivers are fully reimbursed for all extra costs, especially for life-saving treatments such as antibiotic prophylaxis and vaccinations.

*“International guidelines recommend broad meningococcal vaccination for children with sickle cell disease due to their functional asplenia. As you know, they* [children with SCD] *are at much higher risk* [compared to healthy children] *for meningococcal disease. Unfortunately, these vaccines are not covered by health insurance. And for most parents, €25,-* [the price of a vaccine] *is simply too much. Now, we provide the vaccines in the hospital budget, but this simply cannot go on forever.”*

*“Sometimes a certain medication is all of a sudden not covered anymore by an insurance company. For example, for oral penicillin suspension* [essential for infants who cannot take tablets]*, suddenly a very high personal contribution was necessary. We* [paediatric haematologists] *spent many hours together with the clinical pharmacist in order to solve this problem and to avoid these extremely high extra costs for patients. Fortunately, we were able to find a fully covered generic variant which could be imported from a neighbouring country.”*

*“To me, this is an issue of equal access to essential health services for all Dutch citizens. I cannot believe that in a developed country such as the Netherlands, we obligate parents to pay for their child’s much-needed care.”*

*“Apart from this I think access to healthcare is equivalent to access to medication and this is often difficult, as patients are obligated to pay additional fees for various medication types.”*

Due to centralised sickle cell care, some families face high costs because of travelling large distances. In addition, long travelling times may have implications for caregivers’ jobs, as caregivers are often unable to miss a shift or leave work without financial implications or even loss of employment. Furthermore, many children with SCD have siblings, and there is usually no provision for reimbursement of the costs of their care when the caregivers are expected in the hospital with the child who has SCD. In cases of single parent households, this may be even more difficult.

*“Sometimes nurses at the ward report that parents do not visit their hospitalised child often enough. It makes them a bit annoyed and worried about the child’s social situation. While I understand their worries, I also understand that for some parents it’s not always easy to take unpaid leaves of absence in order to visit their child in the hospital.”*

*“One mother ended up getting fired for missing too many days at work. She was on a fixed-term contract. She told her employer about her child with sickle cell disease. He had never heard of the disease before and said it was not his problem.”*

*“Some parents are already struggling every month to just pay the rent. They cannot afford many trips by public transport to the hospital.”*

*“Well, yes, we have a sort of special fund and then - you have to see of course, because you cannot do it too often. It is an emergency fund - you have to estimate how urgent the need for help is, financially I mean. Therefore, we ask advice from our social worker. She is in charge of the fund. Patients can hand in their tickets and receive a reimbursement of the costs of, for instance, their train ride.”*

Overall, despite universal coverage of medical care in Western countries, family borne costs of children with SCD could seriously affect the family’s disposable income. These additional costs could increase inequality in the accessibility of healthcare between households that can easily afford them and those who struggle to make financial ends meet [34]. Reimbursements from government agencies are often insufficient to cover all costs and reimbursement procedures can be quite complicated, especially for individuals with lower health literacy. Previous studies evaluating the impact and financial costs for caregivers of children with other diseases such as diabetes and paediatric cancer show that risk factors of perceived economic hardship include single parenthood, lower socioeconomic status, and physical distance from the treatment centre [35, 36]. The issue of single parenthood requires special attention, even more so because single-heads of household are common in the SCD population [37]. It is pertinent to recognize that many families struggle to meet the extra financial demands of caring for a child with SCD. Therefore, attention must be given to proactive interventions aimed at addressing all extra costs, including full coverage of medical treatment, support for housework and childcare, and access to charitable funding.

### Theme 2. Reducing the number of hospital visits: clustering of appointments on the same day

SCD requires a versatile and comprehensive treatment protocol with frequent check-ups with healthcare professionals from various medical specialties [38]. In the Netherlands, patients visit their paediatric haematologist twice a year to discuss disease progression, treatment and preventative care. Additional hospital visits include check-ups with a nurse practitioner; examinations like transcranial Doppler ultrasound, echocardiography, and laboratory tests; or appointments with medical social workers or psychologists. Therefore, the patients are burdened with multiple appointments throughout the year. Almost all interviewed professionals mentioned that the frequency of hospital visits can present barriers for optimal treatment and that this might be an explanation for the relatively high no-show rates among the patient population. Apart from practical and financial barriers, high no-show rates were also attributed to the patient’s inability to fully understand what different appointments types entail, and why so many hospital visits are necessary.

*“Many patients fail to show up at one or more of their check-up appointments. I think sometimes appointments are forgotten, but I also feel they have too many appointments throughout the year. Parents do not always understand the necessity of each appointment. They think I have already been there three times this year, I do not really have to attend this time.”*

*“For the majority of our patients, it seems difficult for them to fully understand their illness and that even when they are not facing symptoms of a sickle cell crisis; they still have to check-in regularly.”*

Regular follow-up care is required for children and adults with SCD. When consistently followed by a health provider, some disease complications are avoidable. Patients lose vital opportunities for health monitoring and education when regular follow-up appointments are missed, increasing the risk of hospitalisation or mortality.

*“Recently, I saw a female patient of 23 years old who missed her check-ups of the last few years because she had few complaints. Well, now she has lost her sight in one eye, and there is nothing we can do. Even patients with few crises* [vaso-occlusive crises] *and few health issues can develop serious organ damage.”*

A recurring remark in the interviews was the idea that scheduling visits to various healthcare professionals on the same day may be beneficial for total accessibility of care. Not only can this reduce the burden of traveling, it might also become easier to involve additional (para) medical experts such as psychologists to improve comprehensive treatment.

*“Appointments on the same day also make it easier to organise treatment more holistically; for example, adding a visit to a psychologist and a physiotherapist without obliging the family to visit the hospital more often.”*

One participant saw an additional benefit for patients if multiple appointments were offered on the same day. During visits, the intervals between appointments could provide an opportunity for caregivers and patients to meet with other patients and their families.

*“Scheduling visits on the same day could offer an opportunity for children and their families to see and meet fellow sufferers, which could bring the relief of sharing the burden.”*

Lessons in this regard can be drawn from care for children with cystic fibrosis, which is often organised in annual assessment days. On these days, patients and their families speak to a number of healthcare professionals including the specialised paediatrician, other medical specialists, the nurse practitioner, pharmacist, dietician and psychologist. In addition, multiple tests are conducted, such as imaging and lung function tests. Applying this approach in comprehensive SCD centres would address different barriers of healthcare accessibility and thereby help patients and their families to see all required specialists [39, 40].

### Theme 3. Specialised and shared care: bridging the gap

Although care for patients with SCD is centralised, many families still visit their local hospital, because of large travelling distances to the comprehensive sickle cell centre. Almost all participants reported a knowledge gap with regard to SCD among primary care physicians and general paediatricians in local hospitals due to a lack of clinician training and continuing education.

*“Parents told me they took their child with a fever to the general practitioner and he said ‘don’t worry, it’s just a fever. She will get better in a few days; she doesn’t need any prescription medication’. By the time they arrived at my hospital, she* [the child] *had to be rushed into the ICU* [intensive care unit] *with a sepsis. I feel that the risk of bacteraemia and the need for prompt evaluation and treatment is a basic feature of sickle cell disease care.”*

*“It regularly happens that a patient with a crisis* [vaso-occlusive crisis] *visits the general practitioner with severe pain and that he or she then tells them to just take some paracetamol and then they’ll be good to go.”*

*“General practitioners generally have a lack of knowledge of sickle cell disease, but in my experience, they are quite quick with their referral to a haematologist. I feel there is a bigger issue with haematologists in local hospitals. “Because he will think he can handle the patient and doesn’t recognise the seriousness of the disease?” “Yes, that’s what I think.”*

However, some participants shared that they had an excellent working relationship with so-called ‘shared care hospitals’. Shared care is an arrangement between a sickle cell centre and a local hospital or general practitioner.

*“Paediatricians in our shared care hospital are educated to treat children with sickle cell disease. We* [specialised paediatric haematologists in a sickle cell centre] *support and supervise these local healthcare professionals. Whenever a patient does not respond to routine therapy or when there are complications, the patient is transferred to our centre. Communication is very effective.”*

Many participants felt that shorter commutes to the local hospital would notably improve the compliance with attendance at outpatients’ clinics, especially when compared to the – often longer- journey to the sickle cell centre.

*“Some patients travel more than 1.5 hours with public transport to reach our sickle cell centre. Local hospital visits – with consequently much less disruption to the child and family’s everyday routine and without compromising quality – are, for me, an essential part of delivering good healthcare.”*

Participants recommended identification of paediatricians in local (shared care) hospitals with an interest in SCD who could serve as a primary contact with the paediatric haematologist in the centralised sickle cell centre and who are able to disseminate knowledge to other local health professionals when needed.

*“Shared care is about creating a comfortable working relationship between paediatricians and paediatric haematologists. If, for example, all our* [in the sickle cell centre of the participant] *inpatients beds are full and I have a child in the ED* [emergency department] *with a crisis who needs IV* [intravenous] *pain medications, I call the shared care paediatrician with sickle cell disease expertise to discuss the possibility of transferring the patient. I know the child will be in good hands because they know how to treat a child with a crisis* [vaso-occlusive crisis]*, and they will supervise nurses and other hospital workers.”*

In the specific case of migrant children with SCD, several interviewees highlighted that the transfer from one temporary shelter centre to another can be counterproductive to treatment efforts. The geographical location of the shelter determines which general practitioner shared care centre and specialised SCD centre a patient has access to. A transfer to another centre, therefore, often means all healthcare professionals involved in treatment are replaced. Unlucky children switch between medical facilities multiple times during their asylum procedure and receive care from many different healthcare professionals.

*“Children and families in asylum centres are often transferred to other centres across the country. Sometimes I see a patient for the first time, I order laboratory tests, and make a treatment plan, but the next consultation the patient does not come as he or she has been transferred to another centre. That I think is very distressing.”*

*“The asylum centres are extremely badly organised. Caregivers have to take a lot of hurdles to make progress* [ … ] *plus you don’t have your own doctor, so that’s really difficult.”*

Centralised, comprehensive SCD centres have shown to significantly decrease morbidity and to improve quality of life in patients with SCD [41, 42]. However, unfamiliarity with patients with SCD outside these specialised centres makes the patients more likely to receive inadequate care. Simultaneously, sole access to follow-up appointments, emergency care, and inpatient care in the specialised sickle cell centres can be a burden for families living at a large distance from a comprehensive centre. Shared care constructions have been applied in the management of paediatric patients with many (chronic) conditions such as diabetes, cystic fibrosis, idiopathic arthritis, and cancer and is based on a closed collaboration between general paediatricians and specialised paediatricians in centralised centres [43–47]. The shared care hospitals are linked with the specialised centre by a two-way referral and communication process. There are many theoretical benefits in terms of access and convenience. The overall goal is to deliver specialised services as close as possible to the patient’s home without compromising quality. In the case of SCD, primary healthcare providers, including general practitioners, should be supported to improve their knowledge and understanding of SCD. Furthermore, shared care centres should have at least one paediatrician with interest and expertise in SCD and be able to treat mild complications, including vaso-occlusive crises requiring intravenous opioid pharmacotherapy as well as simple infections. Lastly, with special reference to children with SCD in shelter centres, it is important that these children are visible in the healthcare system and are able to be seen regularly by a healthcare professional with knowledge of their disease.

### Theme 4. Optimizing methods of verbal and written communication: enabling mutual understanding between patients and healthcare professionals

Patients with SCD and their caregivers must perform a variety of tasks requiring adequate healthcare understanding, including communication with healthcare professionals, reading and understanding of health information, interpretation of acute symptoms, administration of medication, and making decisions regarding treatment options. Many parents of children with SCD are from ethnic and racial minority groups. Understanding critical information is particularly difficult with a language barrier. Most healthcare professionals interviewed felt that the available health information materials were often hard to read and that caregivers of children with SCD could benefit from having appropriate educational materials about SCD.

*“During the first consultation, we provide parents with an extensive, comprehensive guide to sickle cell disease. It has excellent information; however, I think that for a person without any medical background, it is very hard to understand.”*

Participants reported a limitation in methods to confirm caregiver/patient understanding.

*“When I speak to them they always nod politely but do they really understand what I am saying?”*

Several participants noted a lack of written health information in multiple languages primarily spoken by the patient population such as English and French.

*“The mother was unable to read Dutch, and I was unable to provide any written materials in French.”*

One centre created a visual decision-making educational tool as an aid to enhance communication between the physician and caregiver/patient during the decision-making process of initiation of hydroxyurea therapy.

*“Before* [the educational tool was developed] *I could only provide parents with the pharmacy leaflet on hydroxyurea. That leaflet is really very “scary”; it contains a long list of possible side effects. And the font size is quite small, which makes it more difficult to read. Now I use the visual tool, and I feel they* [the caregivers] *understand the necessity of the treatment much better, and it is easier to address safety concerns.”*

Clear communication and accessible healthcare information is an important component to improve population health [48, 49]. The WHO stresses the importance of understandable health information, reiterating the right of individuals to have access to health information and health systems that they are able to understand and navigate [50]. In addition, special consideration should be given to the development of educational materials for population groups with well-documented low literacy skills, i.e. members of minority population groups and members of immigrant populations.

### Theme 5. Building strong digital connections: improving the use of eHealth and telemedicine

*The interviewed healthcare professionals described the paradoxical ease with which caregivers handle their smartphone, while their low literacy competence interferes with fully comprehending, for example, an appointment letter from the hospital. Making use of a smartphone instead of written letters can improve communication between healthcare professionals and their patients.*

*“Since we started inviting patients for their appointments by e-mail, text message and by admission letters instead of admission letters alone, our no-show rates have declined significantly. Also, it is much easier to remind patients one or two days in advance of the scheduled date.”*

Almost half of the comprehensive sickle cell centres have established a mobile phone number by which caregivers and patients are able to directly call the sickle cell nurse practitioner. During office hours, this number, which bypasses the front desk of the hospital, facilitates a direct link between patients and the healthcare professional. The interviews suggested that caregivers’ preference is to call the nurse directly when requiring support.

*“In contrast to the general hospital phones, our mobile number does not call anonymously. Patients can see it is the sickle cell centre – and not a debt collector for example – who calls them, which increases the chances they pick up the phone. We also use WhatsApp, which works even better than calling. To these messages, we often receive a response almost instantly, while phone calls are sometimes not answered or returned.”*

*A direct mobile phone number supports not only communication through phone calls, but it also enables the exchange of written and spoken communication using* widely used day-to-day messaging applications*. Three interviewees mentioned that the option of spoken messages seem to be particularly useful for caregivers with limited health literacy as no reading or writing is required.*

*“Some parents always contact me by voice message. They send voice memos with questions and concerns like; “when is my child’s next follow-up?”, if they need a new prescription, or when their child is not well. I feel this works really well and lowers the barrier of access to a healthcare professional.”*

Another advantage of direct calls to the sickle cell nurse practitioner is that patients and their caregivers know whom they can call for advice. They can call as soon as they feel the need to, thereby preventing the worsening of their child’s condition.

*“If I explain during a regular follow-up consultation, what to do in case of a vaso-occlusive crisis, it can be difficult for parents to both comprehend and store the information for later use. In case of a stressful event like a painful crisis, it can be very helpful to talk to someone you know and who can give you instructions.”*

However, some healthcare professionals mentioned the specific challenge how to provide caregivers with such a direct line of communication outside working hours.

*“Some caregivers do not really understand that they can only call the sickle cell phone during working hours. In the beginning, I worried caregivers would not know whom to call in case of an emergency outside office hours, so I sometimes answered my phone outside working hours. Currently, I turn my phone off and have a voicemail which provides the phone number of the emergency department.”*

Participants mentioned the increased use of eHealth such as mobile applications to monitor and manage health symptoms and an online portal to access personal medical records. However, this necessitates a certain level of digital health literacy.

*“We send quality of life questionnaires to caregivers’ e-mail addresses one week before the follow-up appointment of their child. Unfortunately, some caregivers never fill in those electronic questionnaires; I feel some don’t really have the skills to use digital technologies.”*

Accessible mobile contact between the SCD nurse practitioner and caregivers can increase caregivers’ capability to manage their child’s care. The use of eHealth services provides a successful way of helping patients to live more optimally with chronic conditions [51]. However, innovative technologies should to be tailored to users’ health literacy skills, which often seems to be forgotten. Otherwise, these technological healthcare innovations may further increase disparities between patients rather than bridge them [52].

### Theme 6. The patient in context: towards compassion and public awareness and a supportive environment

Children with SCD benefit from preventative measures, which include daily use of prophylactic antibiotics, immunisations, ensuring adequate hydration by drinking plenty of fluids, the wearing of warm clothing to avoid chilliness and sufficient rest and avoidance of excessive stress. Although these measures do not seem difficult to safeguard, in a paediatric setting, their success depends heavily on the support a child receives from family, teachers, sports coaches, and many others. Multiple interviewees highlighted that a societal lack of knowledge about SCD often interferes with effective preventive treatment.

*“Some teachers do not allow children to drink from a bottle of water outside of the designated snack- and lunch breaks. This can be a big issue for patients and their families because they may be too shy to inform about the illness or simply not vocal enough to express the children’s needs to drink regularly.”*

Participants described the benefit of a social worker in the comprehensive care team who helps caregivers with the educational system. The social worker can, for example, educate school representatives or can attend school meetings. Keeping in close contact with the school of each patient proved to be an effective approach to increase awareness for better adherence with preventive measures.

*“When a child enters primary school, our social worker always plans a phone call with the teacher of the child to describe the child’s medical needs. We feel this helps enormously in preventing crises because the teacher then understands how to help the child stay safe.”*

*“We use a ‘checklist’ to help parents prepare and remind them of what they need to discuss with their child’s teacher, such as emergency phone numbers, signs or symptoms of pain, fever and fatigue.”*

Increasing general knowledge among key stakeholders and the public is of importance to ensure that preventive and acute healthcare measures are taken in all settings. The participants mentioned the following parties as key stakeholders: the government, municipalities, hospitals and general practitioners (Theme 3), schools, and government authorities in charge of migrants and refugees. Community outreach and educational initiatives would be an important step to inform key stakeholders and society as a whole about the severity and impact of SCD.

*“When I tell people about my work with children with sickle cell disease, many claim they have never heard about the disease.”*

*“I am always surprised when people know about CF [cystic fibrosis] but not about sickle cell disease. Patient numbers in the Netherlands are the same. I don’t understand.”*

Despite the major advances in treatment that have occurred over the past three decades, SCD remains a life-threatening disease that is associated with reduced quality of life. Broader societal awareness of the severity of SCD will increase the likelihood of future government and private financial support for research and the provision of comprehensive and tailored high-quality clinical care.

## Discussion

When evaluating the performance of healthcare systems, national averages of performance indicators fail to acknowledge the individual child’s rights as stated in the United Nations Convention on The Rights of the Child [28]. To complement current knowledge on healthcare accessibility in a high-income country, we performed a nationwide case study among Dutch healthcare professionals in the field of paediatric SCD. This qualitative study explored the intersecting vulnerabilities faced by patients and their families and how these vulnerabilities hamper access to healthcare. Rather than solely identifying barriers, best practices and lessons learnt were gathered from daily clinical practice, supported by existing evidence in the literature.

**Fig. 2:**
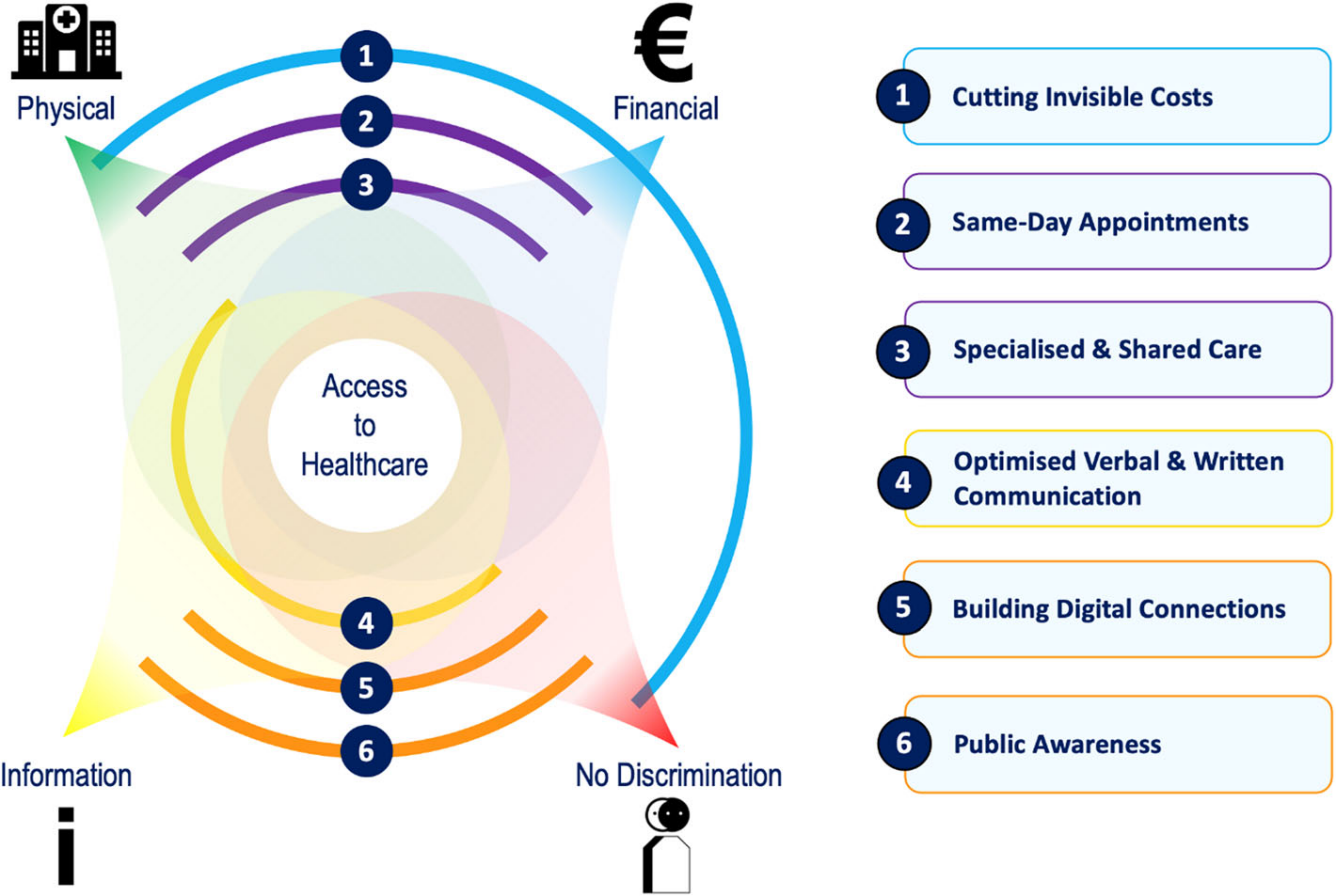
Six key themes crosscutting the four dimensions of healthcare access

Content analyses of the interviews with healthcare professionals revealed six themes with corresponding recommendations (Fig. 2). Together, the recommendations act on all four dimensions of healthcare accessibility: physical accessibility, financial affordability, accessible information, and no discrimination. Most recommendations fall into two or more dimensions of healthcare accessibility. For example, patient appointment reminders by mobile phone instead of long or complicated appointment letters improve the accessibility of health-related information. In addition, in line with the non-discrimination principle, clear communication with patients regardless of their perceived health literacy skills prevents inequality in access between patient groups with different levels of education.

Six themes emerged, all associated with best practices on topics related to improvement of accessibility of healthcare for children with SCD and their families. Firstly, cutting of invisible costs by fully reimbursing caregivers for all extra costs related to the disease of their child. Secondly, clustering of appointments on the same day to help patients seeing all required specialists without having to visit the hospital frequently. Thirdly, improving shared care in order to deliver specialised services as close as possible to the patient’s home without compromising quality. Fourthly, optimising methods of verbal and written communication with special consideration for families with language barriers and/or low literacy skills. Fifthly, improving the use of eHealth services tailored to users’ health literacy skills including accessible mobile telephone contact between healthcare professionals and caregivers of children with SCD. Finally, increasing knowledge and interest in SCD among key stakeholders and the public to ensure that preventive and acute healthcare measures are understood and safeguarded in all settings.

Implementing any of the discussed best practices could lead to an overall improvement of healthcare accessibility. A holistic implementation of all six themes is necessary to adequately address the intersecting vulnerabilities faced by patients with SCD and their families. Some recommendations will be relatively simple to implement. For example, clustering of appointments on 1 day or developing easier to read appointment letters. While such measures are an important step towards improvement of access to care, accessible care cannot be sustained without adequate financial support. For example, the structural improvement of knowledge of SCD among healthcare professionals or the providing of sufficient financial means to cover transportation to the hospital, are more costly.

This qualitative study focuses on the experiences of (mainly white) healthcare professionals and not on caregivers’ or patients’ perceived barriers to accessibility of healthcare. Future studies on caregivers’ perception will be an important extension to the results of this study [53]. In addition, follow-up (quantitative) studies might provide an even stronger foundation for future interventions to improve accessibility of healthcare. For example, how many families exactly face financial hardship? These quantitative studies are ongoing in the Netherlands in the context of the nationwide Dutch research consortium SCORE.

The small-targeted sample in this study, although characteristic for qualitative research, limits the extent to which the findings reported can be generalised to other countries and healthcare systems. Nevertheless, the validity of this multicentre study is supported by the representative sample of healthcare professionals with different occupations caring for children, the internal coherence of the themes and its coherence with the background literature. For now, the six key themes provide recommendations for best practices in the care for paediatric and adolescent patients with SCD and their families. However, medical professionals working outside the field of (paediatric) SCD may recognise that some of their patients face similar barriers in accessing healthcare. Therefore, the recommendations we propose may be worthwhile to implement in other contexts as well.

## Conclusion

This study presents the first overview of both the urgency and the possibility to improve healthcare accessibility for young patients with SCD from the perspective of healthcare professionals. Converged into six key themes, our analysis sheds light on barriers and potential solutions to accessing healthcare, which may serve as a clinically useful resource to improve care for patients with SCD.

## Data Availability

The datasets used and/or analysed during the current study are available from the corresponding author on reasonable request.

## Abbreviations

SCD: Sickle cell disease
WHO: World Health Organisation
ICESCR: International Covenant on Economic, Social and Cultural Rights
CESCR: Committee on Economic, Social and Cultural Rights
HAQ: Healthcare Access and Quality
SRQR: Standards for Reporting Qualitative Research
SCORE: Sickle Cell Outcome Research
